# Rhamnocitrin Attenuates Ovarian Fibrosis in Rats with Letrozole-Induced Experimental Polycystic Ovary Syndrome

**DOI:** 10.1155/2022/5558599

**Published:** 2022-05-26

**Authors:** Yanyuan Zhou, Huan Lan, Zhewen Dong, Wanying Li, Bo Qian, Zhen Zeng, Wen He, Jia-Le Song

**Affiliations:** ^1^Department of Analytical Chemistry & Drug Analysis, School of Pharmacy, Guilin Medical University, Guilin, 541004 Guangxi, China; ^2^Department of Nutrition and Food Hygiene, School of Public Health, Guilin Medical University, Guilin, 541004 Guangxi, China; ^3^State Key Laboratory of Molecular Vaccinology and Molecular Diagnostics, School of Public Health, Xiamen University, Xiamen, 361102 Fujian, China; ^4^Department of Maternal and Child Health, Xiangya School of Public Health, Central South University, Changsha, 410078 Hunan, China; ^5^Department of Clinical Nutrition, The Second Affiliated Hospital of Guilin Medical University, Guilin, 541199 Guangxi, China; ^6^Guangxi Health Commission Key Laboratory of Entire Lifecycle Health and Care, Guilin Medical University, Guilin, 541100 Guangxi, China; ^7^Guangxi Key Laboratory of Environmental Exposureomics and Entire Lifecycle Health, Guilin Medical University, Guilin, 541100 Guangxi, China

## Abstract

Polycystic ovary syndrome (PCOS) is a common endocrine-related cause of infertility in women and has an unknown etiology. Studies have shown that rhamnocitrin (Rha) exhibits positive effects on the reproductive system. This study investigated Rha's antifibrotic effects on PCOS rats and revealed its underlying mechanisms. Female SD rats were randomized into 4 groups (*n* = 8, each); the control group received tea oil by intraperitoneal injection and 1% *w*/*v* CMC by oral gavage; the PCOS group received letrozole (1 mg/kg); the PCOS+Rha group received letrozole and Rha (5 mg/kg); the PCOS+Met group received letrozole and Met (265 mg/kg) for 21 days. At the study end, Rha treatment restored letrozole-induced alterations in the relative ovarian weights, body weight, and relative weights of uterine and visceral adipose tissues. Histological observation showed that Rha ameliorates ovarian structure and fibrosis in PCOS. Administration of Rha reduced letrozole-induced metabolic dysfunction by ameliorating the levels of TC, TG, and HDL-C in the PCOS rats. Rha treatment also modulated the serum levels of sex hormones, which decreased T, E2, and LH and increased FSH in PCOS rats. In addition, Rha treatment modulated insulin resistance and increased gene expression of antioxidant enzymes (Cat, Sod2, Gpx3, Mgst1, Prdx3, Gsta4, Gsr, and Sod1) in the ovaries of the PCOS rats. Finally, Rha treatment appeared to increase the activity of PPAR-*γ* and inhibit the TGF-*β*1/Smad pathway in the ovaries of the PCOS rats. Our findings suggest that Rha significantly ameliorated metabolic disturbances and ovarian fibrosis in the PCOS rats. Rha perhaps is an effective compound for preventing ovarian fibrosis in the future.

## 1. Introduction

Polycystic ovary syndrome (PCOS) is a multifactorial endocrine and metabolic disorder, with a 6-20% prevalence rate in reproductive-age women [1]. This condition is mainly characterized by hyperandrogenism, insulin resistance (IR), chronic oligo- or anovulation, ovarian fibrosis, obesity, and atherosclerotic dyslipidemia [2]. Some risk factors of PCOS have been studied, including lipid imbalance, oxidative stress, IR, diet, and genetic factors [3, 4]. However, the definitive cause is still unclear.

Oxidative stress injury and TGF-*β*1/Smad signal transduction have been widespread in PCOS [5]. Ovarian injury caused by oxidative stress can trigger ovarian fibrosis and eventually lead to a functional decline in the ovary [6, 7]. Furthermore, the main characteristics of PCOS are associated with ovarian fibrosis [8]. PCOS patients have a high level of serum-transforming growth factor-beta 1 (TGF-*β*1), a vital contributing factor in ovarian fibrosis [9]. TGF-*β* binds to TGF-*β*R2, which recruits and phosphorylates TGF-*β*R1 [10]. Active TGF-*β*R1 phosphorylates Smad2/Smad3, which then complex with Smad4 [11]. The complexes bind to gene promoters to induce transcription of profibrotic molecules, such as *α*-smooth muscle actin (*α*-SMA), collagen I, and connective tissue growth factor (CTGF), which increase the synthesis and accumulation of the extracellular matrix [12]. Inhibiting the TGF-*β*1/Smad pathway can effectively improve tissue and organ fibrosis [13]. Improving the endocrine level of PCOS patients, preventing ovarian fibrosis, and restoring normal ovarian function are essential clinical treatment strategies for PCOS [14].

The insulin-sensitizer metformin (Met) and Diane-35 (cyproterone acetate/ethinyl estradiol) are widely used for PCOS patients and can modulate metabolism and hormonal and reproductive activity [15]. In addition, Met has side effects, including renal insufficiency, lactic acidosis, and gastrointestinal disturbances, which can lead to discontinuation of the drug [16].

Currently, plant extracts and natural polyphenols have the potential competence of improving female metabolic and reproductive disorders due to their free radical scavenging properties and chemoprotective ability [17, 18]. Rhamnocitrin (Rha, kaempferol-7-O-methylether) is a flavonoid compound extensively present in *Astagali radix*, *Nervilia fordii*, *Oxytropis ochrocephala Bunge*, *Phyllanthus urinaria*, and *Populus davidiana* [19, 20]. Modern pharmacological studies have demonstrated that Rha has antioxidant, antiatherogenic, anti-inflammatory, and antitumor activities [21]. Our previous study also reported that Rha-enriched total flavonoids extracted from *N. fordii* could improve the endocrine level and reduce the phosphorylation of signal transducer and activator of transcription 3 and Janus kinase-2 in PCOS rats [22]. But, there is no study about Rha's antifibrotic effects on PCOS.

Letrozole, a nonsteroidal aromatase inhibitor, leads to the low conversion of testosterone (T) and androstenedione to estrogens, resulting in the accumulation of androgens [23]. Studies have shown that rats which received letrozole (1 mg/kg) for 21 days can induce PCOS model rats [24, 25]. Compared with other modeling methods, letrozole modeling increased body weight significantly, which was suitable for obesity models. Moreover, letrozole modeling had a high androgen level, consistent with human PCOS characteristics. In addition, oral gavage is less traumatic to rats and easy to operate. The study is aimed at investigating the beneficial effects of Rha on ovarian fibrosis, IR, and dyslipidemia in letrozole-induced PCOS rats.

## 2. Materials and Methods

### 2.1. Chemical Reagents

Rhamnocitrin was obtained from Weikeqi Biotech Co., Ltd. (Sichuan, China). Met (purity more than 98%), carboxymethyl cellulose (CMC), hematoxylin and eosin (H&E), Papanicolaou staining, and Masson's trichrome reagents were acquired from Servicebio Biotechnology Co., Ltd. (Wuhan, China). Letrozole was obtained from Solarbio Life Science Co., Ltd. (Beijing, China). Radio immunoprecipitation assay (RIPA) buffer, BCA assay kit, anti-Smad2 (AF1300), anti-Smad3 (AF1501), anti-Smad4 (AF1291), anti-p-Smad2 (AF2545), anti-p-Smad3 (AF1759), and anti-TGF-*β*1 (AF0297) were obtained from Beyotime Biotechnology Co., Ltd. (Shanghai, China). Anti-CTGF (sc-101586) and anti-Smad7 (sc-365846) were obtained from Santa Cruz Biotechnology, Inc. (Dallas, TX, USA). Anti-*β*-actin (GB11001), anti-PPAR-*γ* (GB11164), anti-*α*-SMA (GB11002), anti-collagen I (GB110022-2), and anti-TGF-*β*R1 (GB11271) were purchased from Servicebio Biotechnology Co., Ltd. The ELISA kits for follicle-stimulating hormone (FSH) (MM-70867R1), testosterone (T) (MM-21165R1), testosterone (T) (MM-0577R1), estrogen (E2) (MM-0575R1), and insulin (INS) (MM-0587R1) were obtained from Meimian Biological Technology Co., Ltd. (Jiangsu, China). Other ELISA kits for triglyceride (TG) (ADS-W-ZF013), total cholesterol (TC) (ADS-W-ZF014), high-density lipoprotein cholesterol (HDL-C) (ADS-DC-348), malondialdehyde (MDA) (ADS-W-YH002), superoxide dismutase (SOD) (ADS-W-KY011), and fasting blood glucose (FBG) (ADS-DC-353) were obtained from Aidisheng Biological Technology Co., Ltd. (Jiangsu, China). All ELISA kit tests are performed according to the manufacturer's guidelines. All other chemicals were of analytical grade.

### 2.2. Experimental Design

Female rats (Sprague-Dawley) aged 6 weeks were acquired from Hunan Silaikejingda Laboratory Animal Co., Ltd. (Changsha, Hunan, China). The rats with a weight of 190-220 g were caged in a standard specific pathogen-free (SPF) environment of (23 ± 3°C, 12 h light/dark) and *ad libitum* fed standard AIN-93M diet and water. After acclimatising 7 days, the rats were divided into 4 groups (8 rats/group) designated the control, PCOS, PCOS+Rha, and PCOS+Met groups. The control group received medicinal camellia oil (Rha vehicle) by intraperitoneal injection and 1% CMC (letrozole vehicle) by oral gavage; the PCOS group was administrated letrozole (1 mg/kg); the PCOS+Rha group received letrozole and Rha (5 mg/kg); the PCOS+Met group was treated with letrozole and Met (265 mg/kg) [26]. The treatments were administered daily for 21 days, and all rats were weighed every 3 days during the experiment. After laparotomy, all experimental animals were euthanized with carbon dioxide. The study was subject to approval by the Institutional Animal Care and Use Committee of Guilin Medical University (IACUC-GMU, approval number: GLMC-201806003).

### 2.3. Vaginal Smears

Vaginal smears were collected daily and determined by the Papanicolaou staining assay. Normal saline-soaked aseptic cotton swabs were placed on the rat vagina, smeared clockwise and on pathological grade slides. After that, the slides were fixed with an ethyl alcohol (95%) solution for 15 min. Finally, the slides were stained according to the kit's guidelines.

### 2.4. Blood Sampling, Oral Glucose Tolerance Test (OGTT), and IR Assay

After the last administration, all animals were first anesthetized with Zoletil 50 (40 mg/kg; Virbac Laboratories, Carros, France), and blood was collected from the caudal vein after overnight fasting. The glucose load and FBG level were measured by an ELISA kit (Aidisheng Biological Technology Co., Ltd.) and denoted as 0 min.

All rats were administered glucose (2 g/kg BW) by oral gavage, and glucose concentrations were measured after 30, 60, 90, and 120 minutes. The area under the OGTT curve (AUC) is used to reflect glucose tolerance [27]. The serum levels of INS were measured by an ELISA kit (Meimian). The IR levels were measured using the formula: HOMA‐IR = FBG (mmol/L) × fasting INS (*μ*U/L)/22.5 [28]. Then, the remaining blood was collected into vacuum blood collection tubes (Solarbio) and centrifuged at 3000 *g* for 10 min in an X-22R refrigerated centrifuge (Beckman Coulter). All collected serum was frozen at -80°C until further use. Finally, the ovary, uterus, heart, liver, spleen, lung, kidney, and visceral adipose tissue (perirenal, abdominal, and periuterine fats) were isolated from the sacrificed rats, and the relative weight (which is indicated as g/BW × 100) was calculated.

### 2.5. Determination of Sex Hormones and Lipid Concentrations

The serum levels of LH, FSH, E2, and T were determined by ELISA kits (Meimian). The serum levels of TG, HDL-C, and TC were measured spectrophotometrically using kits (Aidisheng). The TC/HDL-C and TG/HDL-C ratios were estimated. The triglyceride-glucose (TyG) index can be used to identify subjects with low insulin sensitivity [29]. The TyG index Ln [TG (mg/dL) × FBG (mg/dL)/2] was determined [28].

### 2.6. Determination of MDA and SOD

The serum levels of MDA and SOD were measured by ELISA kits (Kete).

### 2.7. Histopathologic Observation

Ovarian tissue and periuterine fat were fixed in the formaldehyde (10%). Then, tissue samples were embedded, cut into 4 *μ*m cross sections, and stained with H&E and Masson's reagents, respectively. Histological observations were acquired and recorded under the microscope (Leica, Germany).

### 2.8. qRT-PCR Assay

Total RNA was extracted from ovarian tissues using Trizol reagent (Tiangen, Beijing, China). The mRNA levels of target genes were determined using the quantitative PCR reagent kit (Tiangen) in a QuantStudio 6 Flex Real-Time PCR System (Thermo Fisher Scientific, Waltham, MA, USA). The reaction condition and primer sets were as previously described [30].

### 2.9. Western Blotting

Ovaries tissues (40 mg) were quickly removed, washed PBS, shredded and lysed using ice-cold RIPA buffer (0.4 mL), and then broken with a homogenizer (Servicebio) at 4°C. The protein concentrations of tissue samples were determined by a BCA kit according to the manufacturer's instructions. The isolated protein (30 *μ*g) was subjected to SDS-PAGE (10~12%) and transferred to nitrocellulose membranes (Merck, Germany). The membranes were blocked with defatted milk (5%) for 2 h at 37°C and then incubated overnight at 4°C with the following primary antibodies: TGF-*β*1, TGF-*β*R1, Smad2, p-Smad2, Smad3, p-Smad3, Smad4 (1 : 1000), Smad7, CTGF, *α*-SMA, PPAR*γ*, collagen I, and *β*-actin. The dilution ratio of primary antibodies is as previously described [30]. Next, the membranes were incubated with horseradish peroxidase- (HRP-) labeled secondary antibodies (1 : 1000) at 37°C for 1 h and washed twice. The bands were visualized using an enhanced chemiluminescence reagent (Beyotime). *β*-Actin was used as the internal control.

### 2.10. Immunohistochemical (IHC) Staining Assay

The ovarian tissue levels of TGF-*β*1, Smad2, Smad3, Smad4, and Smad7 were employed with an IHC staining assay. The sections (4 *μ*m) were warmed at 37°C overnight in an oven. Then, the sections were dewaxed (by xylene), and the slides were rehydrated (by alcohol) in different gradients. The antigen retrieval process was conducted for 15 min with EDTA antigen retrieval solution (pH 9.0). Endogenous peroxidase activities were blocked by hydrogen peroxide (3%, vol/vol) in PBS for 20 min at 37°C. The nonspecific activity was blocked by 10% normal goat serum (vol/vol) for 30 min at 37°C and then incubated with the primary antibodies. Then, the slides were incubated with streptavidin-HRP solution for 20 min and incubated with a diaminobenzidine solution for 10 min at 37°C. Finally, hematoxylin was used for counterstaining. Positive IHC staining appeared brown.

### 2.11. Statistical Analysis

The data were shown as mean ± standard deviation (SD) and analyzed by SPSS 18.0 software. Significant differences between the two groups were determined by one-way analysis of variance (ANOVA) with the post hoc Bonferroni test. A *P* value < 0.05 was considered statistically significant.

## 3. Results

### 3.1. The Irregular Estrous Cycle of the PCOS Rats

**Figure 1:**
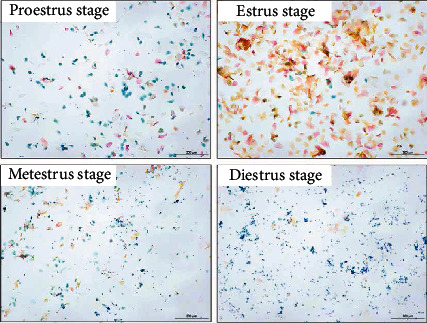
Picture of each estrous cycle in rats. Cellular characteristics of the estrous cycle were determined by Papanicolaou staining assays (×100).

To assess the construction of the PCOS model, vaginal smears were used to monitor the estrus cycle and collected daily. In the control group, the results showed that the rats had a normal estrous cycle, including proestrum, oestrum, metestrus, and diestrum stages (Figure 1). In the PCOS group, the estrous cycle was prolonged and mainly composed of leukocytes. The features confirmed PCOS rat model were established. The PCOS+Rha group and the PCOS+Met group showed a normal estrous cycle when compared to the PCOS group. Our findings suggest that Rha reversed letrozole-induced cycle arrest in PCOS rats.

### 3.2. Effects of Rha on the Histological Findings of the Ovaries, Uterus, and Adipocyte

**Figure 2:**
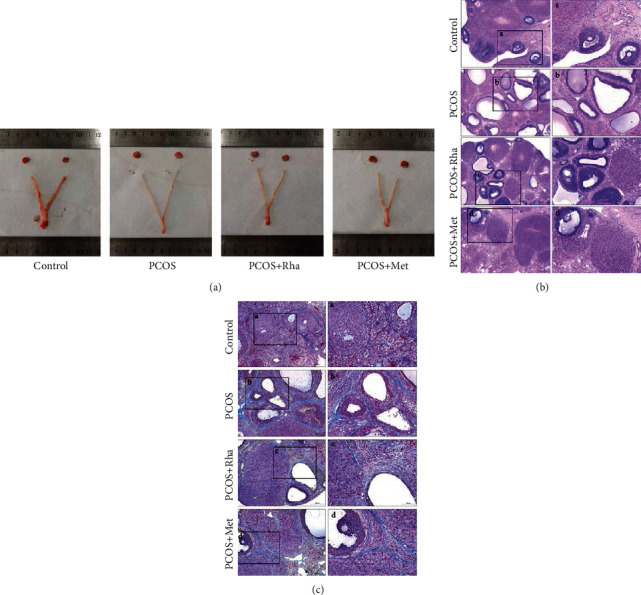
Effects of rhamnocitrin (Rha) on ovarian, uterine, and periuterine adipocyte morphology. (a) Photographs of the morphology of the ovaries and uterus. (b) HE staining of ovarian tissue (scale bar = 500 *μ*m; black box defines the area to be amplified, high magnification scale bar = 20 *μ*m). (c) Masson's trichrome staining of ovarian tissue (scale bar = 200 *μ*m; black box defines the area to be amplified, high magnification scale bar = 100 *μ*m).

The ovaries in the PCOS group were pale and volume increased (Figure 2(a)). The uterus in the PCOS group was pale and had a low blood supply. In contrast, the ovaries and uterus in the control group were bright red and had a greater blood supply. These pathological lesions were improved in the PCOS+Rha group and the PCOS+Met group. The histopathological examination of the ovary tissues was analyzed by light microscopy. Corpora luteum and healthy follicles at different developmental stages were observed in the control group (Figures 2(b) and 2(a). In contrast, the ovaries of PCOS rats displayed cystic expansion, and the number of luteum and healthy follicles was reduced in comparison with that in the control group. Furthermore, the granular cell layers were decreased and atresia follicles were found. Compared to the PCOS group, the PCOS+Rha group (Figures 2(b) and 2(c)) and the PCOS+Met group (Figure 2(b)–2(d)) reduced the number of cystic follicles with an increased thickness of the granulosa cell layer and a decrease in theca cells. In addition, Masson's trichrome staining results showed that ovarian fibrosis was dramatically increased in the PCOS group rats (Figure 2(c) and 2(a) in comparison with the control group. Compared with the PCOS group, ovarian fibrosis was obviously inhibited in the PCOS+Rha group (Figures 2(b)–2(c)) and the PCOS+Met group (Figures 2(c)–2(d)). However, the inhibitory effect was greater in the Rha-treated group compared with the Met-treated group. These results together suggest that Rha ameliorates ovarian structure and fibrosis in PCOS rats.

### 3.3. Effect of Rha on the BW and Visceral Adipose Weight

**Figure 3:**
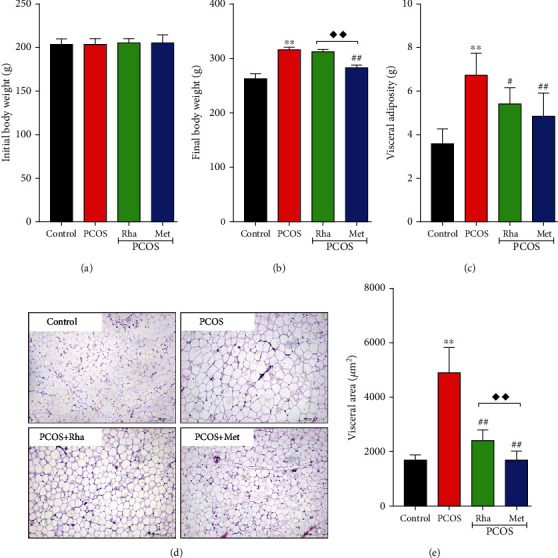
Effect of rhamnocitrin (Rha) on body weight (BW) and visceral adiposity. (a) Initial BW, (b) final BW, (c) visceral adiposity, (d) H&E staining of periuterine adipocytes (scale bar = 200 *μ*m), and (e) adipocytes area. ^∗^*P* < 0.05 and ^∗∗^*P* < 0.01 vs. the control group; ^#^*P* < 0.05 and ^##^*P* < 0.01 vs. the PCOS group; ^◆^*P* < 0.05 and ^◆◆^*P* < 0.01, the PCOS+Rha group vs. the PCOS+Met group. Values are mean ± SD (*n* = 8).

The BW and visceral adipose weight of rats in each group are presented in Figure 3. There was no significant difference between all groups for initial BW. After 21 days, the BW and visceral adipose weight significantly increased in the PCOS group when compared to the control group (*P* < 0.01). Met treatments reduced the BW and visceral adipose weight when compared with the PCOS group (*P* < 0.01). Compared with the PCOS group, Rha treatments reduced the visceral adipose weight (*P* < 0.05), whereas the BW was not statistically altered (*P* > 0.05).

In addition, HE staining was performed on adipocytes (Figure 3(d)). Photomicrographs showed that the adipocyte area was lower in the control group in comparison with the PCOS group (*P* < 0.01). The adipocyte area was significantly decreased in the PCOS+Rha group and the PCOS+Met group compared with the PCOS group (*P* < 0.01). Indeed, the adipocyte area in the PCOS+Met group was lower compared with that in the PCOS+Rha group (*P* < 0.01) (Figure 3(e)). These results indicate that Rha modulates metabolic disturbances in PCOS rats.

### 3.4. Effect of Rha on the Relative Organ Weights

The relative weight data of the organs are shown in Table 1. Compared with the control group, the relative weights of the ovaries were increased (*P* < 0.05), whereas the relative weights of the uterus were decreased (*P* < 0.01) in the PCOS group. Rha treatment and Met treatment significantly decreased (*P* < 0.05 and *P* < 0.01, respectively) the relative weights of the ovaries and remarkably increased (*P* < 0.01) the relative weights of the uterus. The relative weights of the liver have significant loss (*P* < 0.01) in the PCOS group in comparison with the control group.

Treatment with Rha and Met showed no significant effect on the relative weights of the liver compared to the PCOS group (*P* > 0.05). No significant difference was observed regarding the relative weights of the heart, kidney, lung, and spleen among the four groups (*P* > 0.05). These results together suggest Rha has no adverse effects on PCOS rats.

### 3.5. Effects of Rha on the Serum Levels of FBG and Insulin

**Figure 4:**
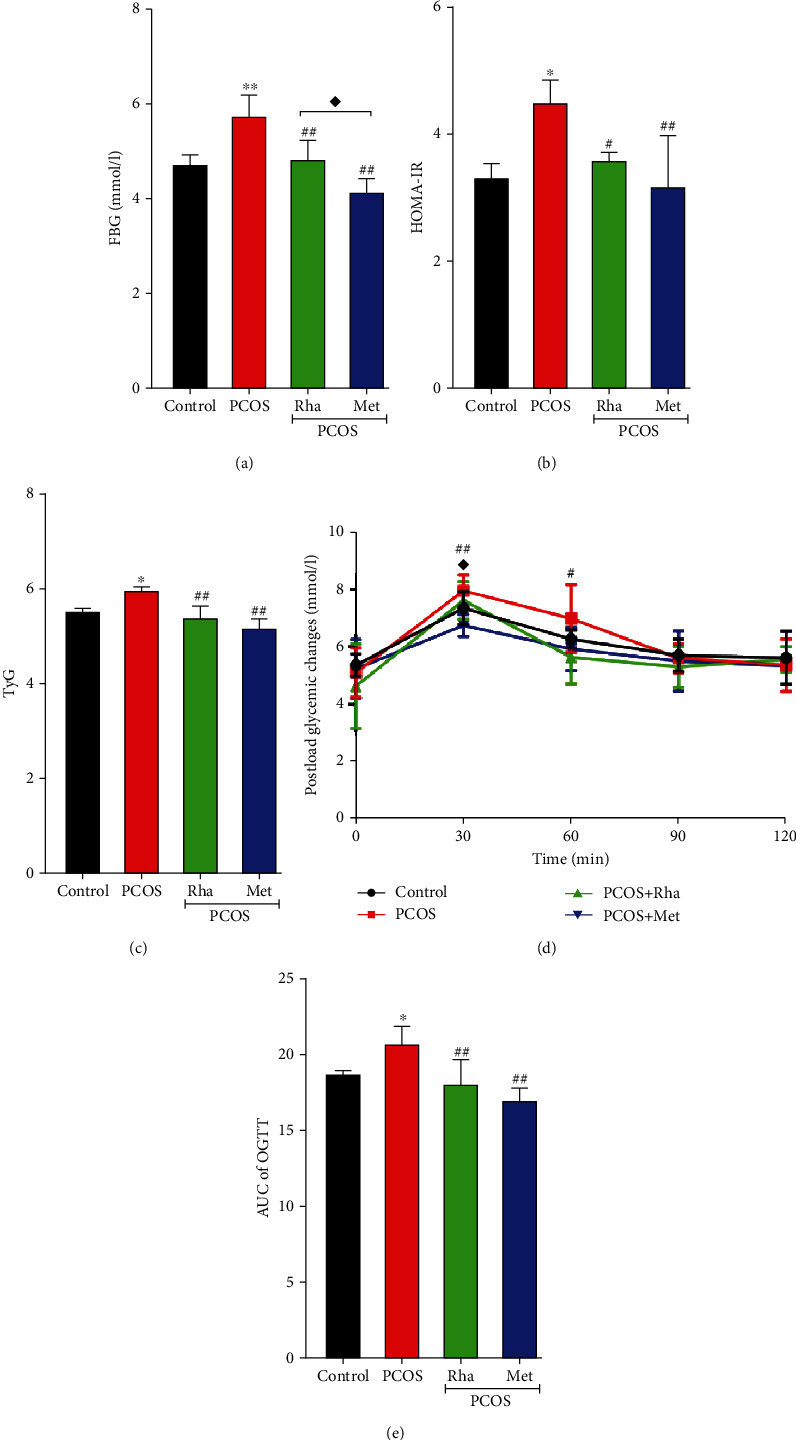
Effects of rhamnocitrin (Rha) on the blood levels of (a) FBG, (b) HMOA-IR, (c) and TyG; (d) postload glycemic change; and (e) AUC of OGTT. ^∗^*P* < 0.05 and ^∗∗^*P* < 0.01 vs. the control group; ^#^*P* < 0.05 and ^##^*P* < 0.01 vs. the PCOS group; ^◆^*P* < 0.05 and ^◆◆^*P* < 0.01, the PCOS+Rha group vs. the PCOS+Met group. Values are mean ± SD (*n* = 8).

PCOS is a severe disease that is associated with IR [31]. The levels of FBG were higher in the PCOS group than in the control group (*P* < 0.01) (Figure 4). In both Rha treatment and Met treatment, remarkable decreases in FBG levels are observed (*P* < 0.01). Furthermore, the Rha-treated group caused more decrease in FBG levels as compared to the Met-treated group (*P* < 0.05). HOMA-IR was markedly increased in the PCOS group in comparison with the control group. HOMA-IR was significantly decreased (*P* < 0.05 and *P* < 0.01, respectively) in the Rha-treated group and the Met-treated group in comparison with the PCOS group. The PCOS group showed markedly elevated TyG levels compared with the control group (*P* < 0.05). Furthermore, both Rha treatment and Met treatment significantly reduced the elevated TyG levels in the PCOS rats (*P* < 0.01). The AUC of the OGTT was increased significantly (*P* < 0.01) in the PCOS group in comparison with the control group. The AUC of the OGTT was obviously decreased (*P* < 0.01) in the Rha-treated group. However, the differences in the AUC of the OGTT were not significant between the Met-treated groups and the PCOS group (*P* > 0.05). We concluded that Rha could improve IR in PCOS rats.

### 3.6. Serum Levels of FSH, LH, T, and E2 in Rha-Treated PCOS Rats

**Figure 5:**
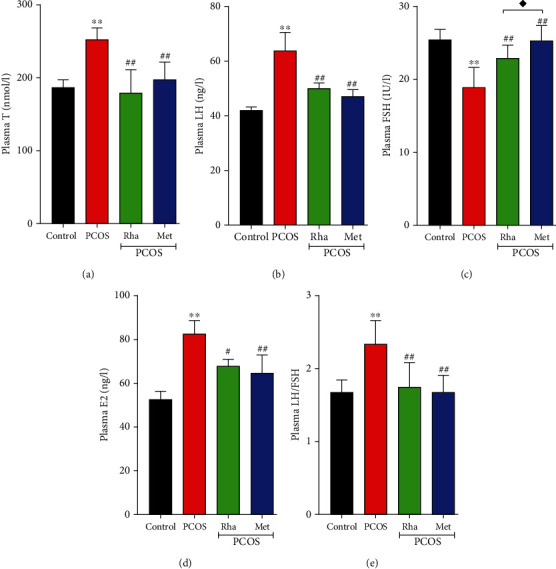
Effects of rhamnocitrin (Rha) on the serum levels of (a) T, (b) LH, (c) FSH, (d) E2, and (e) the LH/FSH ratio. ^∗^*P* < 0.05 and ^∗∗^*P* < 0.01 vs. the control group; ^#^*P* < 0.05 and ^##^*P* < 0.01 vs. the PCOS group; ^◆^*P* < 0.05 and ^◆◆^*P* < 0.01, the PCOS+Rha group vs. the PCOS+Met group. Values are mean ± SD (*n* = 8).

The serum level of sex hormone is a vital index to indicate PCOS in clinical diagnoses and basic studies. Compared with the control group, the serum levels of T, E2, LH, and the LH/FSH ratio were obviously elevated (*P* < 0.01), whereas the levels of FSH were obviously decreased (*P* < 0.01) in the PCOS group. Rha and Met notably decreased the levels of T, E2, LH, and the LH/FSH ratio and remarkably increased the levels of FSH (Figure 5). The levels of FSH (*P* < 0.05) were higher in the Met-treated group in comparison with the Rha-treated group. These results together suggest Rha modulates hormone levels in PCOS rats.

### 3.7. Effect of Rha on the Serum Lipid Profile

**Figure 6:**
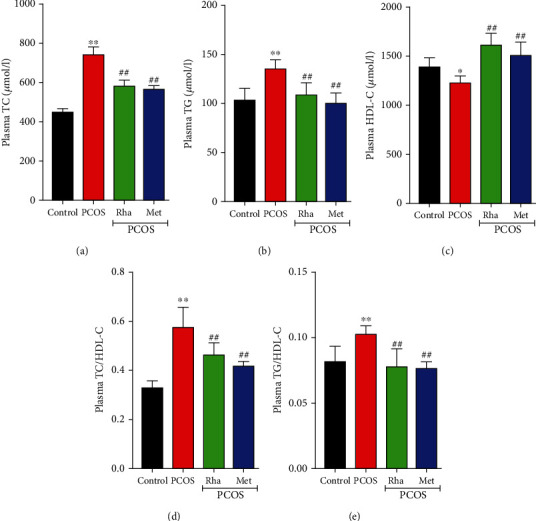
Effects of rhamnocitrin (Rha) on the serum levels of (a) TC, (b) TG, (c) HDL-C, (d) the TC/HDL-C ratio, and (e) the TG/HDL-C ratio. ^∗^*P* < 0.05 and ^∗∗^*P* < 0.01 vs. the control group; ^#^*P* < 0.05 and ^##^*P* < 0.01 vs. the PCOS group. Values are mean ± SD (*n* = 8).

PCOS is a metabolic disease characterized by dyslipidemia. When compared with the control group, serum levels of TC, TG, TC/HDL-C, and TG/HDL-C were dramatically increased (*P* < 0.01), while HDL-C levels were markedly decreased (*P* < 0.01) in the PCOS group (Figure 6). Both Rha treatment and Met treatment obviously reduced (*P* < 0.01) the levels of TC, TG, TC/HDL-C, and TG/HDL-C with a significant increase (*P* < 0.01) in the HDL-C levels (*P* < 0.01) in comparison with those of the PCOS group. We concluded that Rha ameliorated dyslipidemia in PCOS rats.

### 3.8. Serum Levels of MDA and SOD in Rha-Treated PCOS Rats

**Figure 7:**
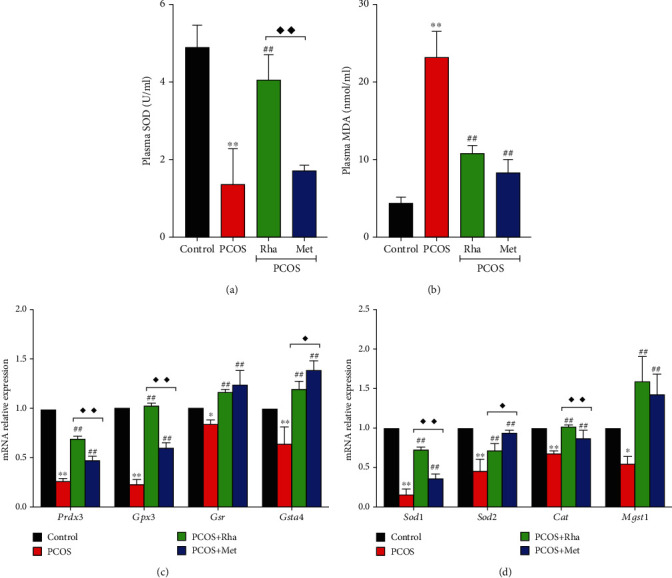
Effects of rhamnocitrin (Rha) on the serum levels of (a) MDA and (b) SOD and (c, d) the mRNA levels of antioxidant enzymes (*Cat*, *Sod2*, *Gpx3*, *Mgst1*, *Gsta4*, *Gsr*, *Sod1*, and *Prdx3*). Values are mean ± SD. ^∗^*P* < 0.05 and ^∗∗^*P* < 0.01 vs. the control group; ^#^*P* < 0.05 and ^##^*P* < 0.01 vs. the PCOS group; ^◆^*P* < 0.05 and ^◆◆^*P* < 0.01, the PCOS+Rha group vs. the PCOS+Met group.

Oxidative stress is associated with PCOS inducing serious tissue damage [32]. As shown in Figure 7, the serum levels of MDA were profoundly enhanced (*P* < 0.01), and the levels of SOD were markedly decreased in the PCOS group (*P* < 0.01). Both Rha and Met treatments significantly decreased (*P* < 0.01) the serum MDA generation in the PCOS rats. Rha treatment obviously increased the SOD levels in the PCOS rats (*P* < 0.01). By contrast, the SOD levels were slightly but not significantly increased by Met treatment.

### 3.9. The mRNA Levels of Antioxidant Factors in the Ovarian Tissues of the Rha-Treated PCOS Rats

The mRNA levels of antioxidant factors (such as Cat, Sod2, Gpx3, Mgst1, Gsta4, Gsr, Sod1, and Prdx3) were decreased sharply in the PCOS group in comparison with the control group (Figure 7). Moreover, administration of either Rha or Met significantly increased the Sod1, Sod2, Cat, Mgst1, Prdx3, Gpx3, Gsr, and Gsta4 mRNA levels compared with those in the PCOS group (*P* < 0.01). Importantly, the mRNA levels of Sod1, Cat, Prdx3, and Gpx3 in the Rha-treated group were obviously higher (*P* < 0.01) than those in the Met-treated group. The mRNA levels of Sod2 and Gsta4 in the Met-treated groups were obviously lower (*P* < 0.05) in comparison with those in the Rha-treated group. These data together suggest that antioxidant plays a vital role in ovaries. Rha treatment could inhibit oxidative stress in PCOS rats.

### 3.10. Rha Treatment Modulated the Activation of the Smad Pathway in the Ovarian Tissues of PCOS Rats

**Figure 8:**
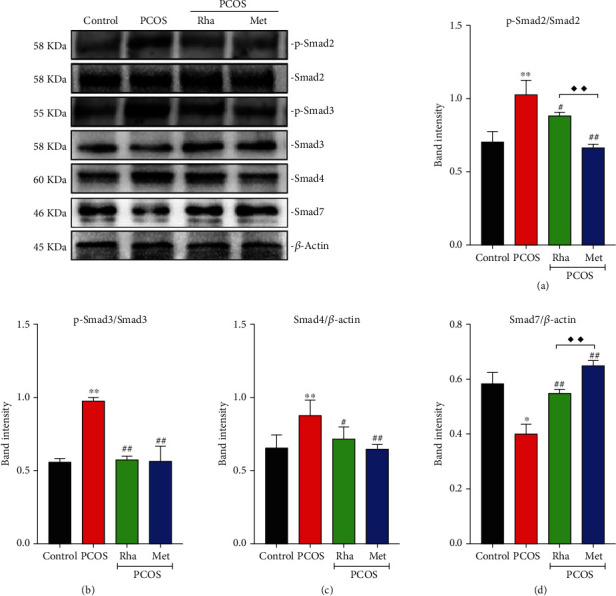
Effect of rhamnocitrin (Rha) on the protein levels of (a) p-Smad2, (b) p-Smad3, (c) Smad4, and (d) Smad7 in the ovary. Values are mean ± SD. ^∗^*P* < 0.05 and ^∗∗^*P* < 0.01 vs. the control group; ^#^*P* < 0.05 and ^##^*P* < 0.01 vs. the PCOS group; ^◆^*P* < 0.05 and ^◆◆^*P* < 0.01, the PCOS+Rha group vs. the PCOS+Met group.

The levels of p-Smad2, p-Smad3, and Smad4 were significantly increased (*P* < 0.01) and the levels of Smad7 were dramatically decreased (*P* < 0.05) in the PCOS group as compared to the control group (Figure 8). Compared to the PCOS group, the levels of p-Smad2 and Smad4 significantly decreased in the Rha-treated group and the Met-treated group (*P* < 0.05 and *P* < 0.01, respectively).

Compared to the PCOS group, the levels of p-Smad3 significantly decreased (*P* < 0.01), while the levels of Smad7 were dramatically increased (*P* < 0.01) in the Rha-treated group and the Met-treated group.

The levels of p-Smad2 in the Met-treated group were lower than in the Rha-treated group (*P* < 0.01). The levels of Smad7 in the Met-treated group were higher than those in the Rha-treated group (*P* < 0.01).

### 3.11. Effect of Rha on the Activity of TGF-*β*1, TGF-*β*R1, *α*-SMA, PPAR-*γ*, Collagen I, and CTGF in the Ovarian Tissues of the PCOS Rats

**Figure 9:**
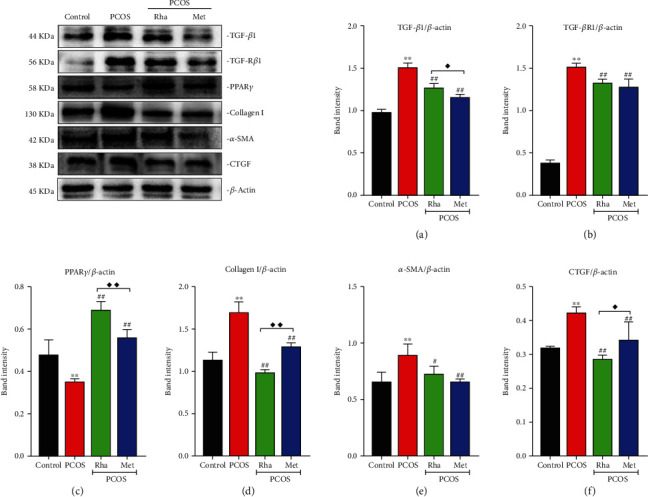
Effect of rhamnocitrin (Rha) on the protein levels of (a) TGF-*β*1, (b) TGF-*β*R1, (c) PPAR-*γ*, (d) collagen I, (e) *α*-SMA, and (f) CTGF in the ovary. Values are mean ± SD. ^∗^*P* < 0.05 and ^∗∗^*P* < 0.01 vs. the control group; ^#^*P* < 0.05 and ^##^*P* < 0.01 vs. the PCOS group; ^◆^*P* < 0.05 and ^◆◆^*P* < 0.01, the PCOS+Rha group vs. the PCOS+Met group.

The levels of TGF-*β*1, TGF-*β*R1, *α*-SMA, collagen I, and CTGF were obviously increased (*P* < 0.01), while PPAR-*γ* was obviously decreased (*P* < 0.01) in the PCOS group in comparison with the control group (Figure 9). The levels of TGF-*β*1, TGF-*β*R1, and collagen I were obviously decreased (*P* < 0.01) and the levels of PPAR-*γ* were dramatically increased (*P* < 0.01) in the Rha-treated group and the Met-treated group in comparison with the PCOS group. The levels of TGF-*β*1 in the Met-treated group were lower than those in the Rha-treated group (*P* < 0.01). The levels of PPAR-*γ* in the Rha-treated group were higher than those in the Met-treated group (*P* < 0.05). The levels of collagen I in the Rha-treated group were lower than those in the Met-treated group (*P* < 0.05). The levels of *α*-SMA were significantly decreased (*P* < 0.05 and *P* < 0.01, respectively) in the Rha-treated group and the Met-treated group in comparison with the PCOS group. The levels of CTGF significantly decreased (*P* < 0.01) in the Rha-treated group and the Met-treated group in comparison with the PCOS group. The levels of CTGF in the Met-treated group were higher compared to those in the Rha-treated group (*P* < 0.05). These results show that Rha could inhibit the activation of the TGF-*β*1/Smad signaling pathway in PCOS rats.

### 3.12. Effect of Rha on IHC Staining of the Ovarian Tissues of the PCOS Rats

**Figure 10:**
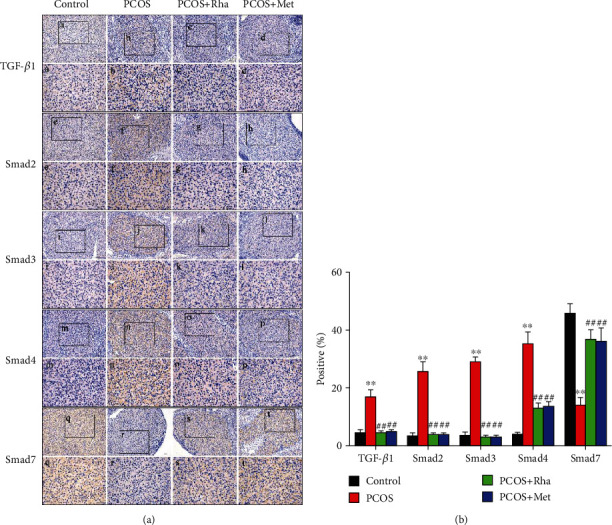
Effect of rhamnocitrin (Rha) on the protein expression of TGF-*β*1, Smad2, Smad3, Smad4, and Smad7 in the ovary. Representative immunohistochemistry images of the TGF-*β*1, Smad2, Smad3, Smad4, and Smad7 proteins in the ovary are shown with quantification (scale bar = 100 *μ*m; black box defines the area to be amplified, high magnification scale bar = 50 *μ*m). ^∗^*P* < 0.05 and ^∗∗^*P* < 0.01 vs. the control group; ^#^*P* < 0.05 and ^##^*P* < 0.01 vs. the PCOS group. Values are mean ± SD (*n* = 8).

The IHC images indicated that the ovarian sections from the PCOS group presented a significant increase in TGF-*β*1, Smad2, Smad3, and Smad4 expression with a significant reduction in Smad7 expression as compared to those from the control groups (*P* < 0.01) (Figure 10). Treatment with either Rha or Met significantly reduced the pixel-based intensity of TGF-*β*1, Smad2, Smad3, and Smad4 with a significant increase in the pixel-based intensity of Smad7 expression compared to that of the PCOS group (*P* < 0.01). Nevertheless, the PCOS+Rha group demonstrated higher Smad7-positive reactions than the PCOS+Met group.

## 4. Discussion

PCOS is a systemic disease with an unknown etiology characterized by metabolic disorder and endocrine disorder. At present, improving the endocrine level of PCOS patients, preventing the occurrence of ovarian fibrosis, and restoring normal ovarian function are the main strategies for clinical PCOS treatment [14]. Many studies have suggested that plant polyphenols can improve metabolism, restore endocrine function, and exhibit a curative effect in PCOS animal studies and clinical PCOS cases [33–35].

In this study, letrozole significantly induced increases in BW, visceral adiposity, and relative ovary weight in the PCOS rats (Figures 3(b) and 3(c)). Obesity, one of the most common diseases in PCOS patients, is associated with complex clinical features, including infertility and menstrual irregularity [1]. Obesity, insulin resistance, oxidative stress, and androgen level have a complex interrelationship in the pathogenesis of PCOS. Several studies have suggested increasing T levels cause metabolic imbalances and weight gain in PCOS [36, 37]. The increased BW is perhaps due to the accumulation of fatty tissue. Besides, the increased relative ovarian weight may be caused by the formation of follicular cysts [38]. In addition to improving metabolism by regulating hormone levels, the underlying antioxidant capacity leads to the reduced fat formation, and reduced follicular cysts (follicular fluid) also have a significant effect on obesity [39]. After Met treatment, the BW and relative ovary weight were significantly decreased in the PCOS rats (Figures 3(b) and 3(c)). It suggests that Met contributes to decrease the fatty formation and follicular cysts. Although Rha did not significantly improve the weight of the PCOS rats, it significantly reduced the relative ovarian weight and visceral adiposity (Figure 3(c)). We also observed that adipocyte sizes were larger in the PCOS group (Figure 3(d)). Increasing evidence suggests that excess androgen enhances adipocyte size in women with PCOS. This adipocyte hypertrophy may lead to adipocyte dysfunction, and the differentiation of hypertrophic adipocytes to smaller adipocytes may be an effective strategy to improve IR and obesity [37]. After Rha treatment, the adipocyte size of the PCOS rats was significantly reduced (Figure 3(d)). The results suggested that Rha could reduce the fat formation and follicular cysts in PCOS rats.

Our results found that T levels were obviously increased in the PCOS rats (Figure 5(a)), which reflects the overexpression of androgen. Previous studies by others have reported that letrozole induces PCOS by blocking the conversion of T to estrogen, subsequently resulting in circulating hyperandrogenism and hormonal imbalance [24, 40]. It has been shown that the hypothalamic-pituitary-gonad (HPG) axis plays a crucial role in regulating reproductive function and hyperandrogenism responsible for abnormal ovarian physiology, owing to the disruption of the HPG axis [41, 42]. PCOS rats exhibited high LH levels, low FSH levels, and an increased LH/FSH ratio, which indicated the disruption of the HPG axis. Similar to this study, the previous studies show that PCOS patients exhibited a significantly increased LH/FSH ratio, which can interfere with ovulation [25]. Moreover, a low level of constant FSH stimulation stops the small follicles in ovarian development, thereby resulting in polycystic ovaries and anovulation in PCOS [43]. In this study, the serum E2 levels were increased in the PCOS rats, as shown by previous studies. Increased E2 levels were correlated with abnormal estrus cyclicity and multiple cyst formation [44]. In this study, the ovaries of PCOS rats displayed cystic expansion and lack of oocytes, and the number of healthy follicles and luteum was reduced. Furthermore, the granular cell layers were decreased and atresia follicles were found in the PCOS rats, as reported by a previous study [25, 45]. The damaged ovarian structure in our histological analysis has confirmed the abnormal sex hormone levels in PCOS rats. There is a strong correlation between ovarian granulosa cell proliferation and follicular growth. Androgen not only promotes the proliferation of granulosa cells and inhibits their apoptosis but also promotes the growth of follicles [40]. Elevated androgen can affect and inhibit follicular development, resulting in polycystic changes in the ovary [46]. In our study, Met treatment improves sex hormone levels and recovers histological alterations of ovaries. It has been reported that Met administration decreased T levels, increased LH/FSH ratio, and induced ovulation in mice with PCOS, suggesting Met can regulate the HPG axis [47, 48]. Furthermore, Rha-treated rats showed decreased T levels and E2 and an increased ratio of LH/FSH have confirmed its regulatory function on sex hormone levels. At the same time, Rha-treated rats showed a marked recovery of ovarian tissue with the appearance of developing and antral follicles, a prominent reduction in cysts, and regular luteinization. Our results suggest that Rha regulates sex hormone levels and improves ovarian morphology by acting on the HPG axis.

Abnormal insulin metabolism in PCOS patients results from defective insulin receptor signaling pathways that impair glucose transfer through insulin [49]. In this study, we observed that the FBG level was significantly increased in the PCOS rats (Figure 4(a)). During the pathophysiological process of PCOS, a state of severe IR induces a decrease in the glucose consumption of the body and stimulates pancreatic *β* cells to release more insulin in order to increase glucose consumption in the muscles [50]. Overgeneration of insulin can activate androgen production in thecal cells of individuals with PCOS [51]. The IR and hyperinsulinemia created by letrozole are related to hyperandrogenism [52]. High circulating levels of T negatively affect IR in PCOS population [53]. We observed that Met significantly decreased the FBG and TyG levels in the PCOS rats (Figures 4(a) and 4(c)), as shown by Jahan's studies [17]. Although Rha was not as effective as Met, it could effectively improve these parameters. Decreased T levels in Met and Rha groups could be the reason for improved IR in these groups. An increase in HOMA-IR, a surrogate marker of insulin sensitivity, as seen in the PCOS group, indicates the development of IR. A decrease in HOMA-IR in the Met groups and Rha groups suggests an improvement in insulin sensitivity (Figure 4(b)). Therefore, we suggest that Rha administration maintaining glucose homeostasis improves IR by modulating insulin secretion and enhancing insulin-mediated glucose uptake.

PCOS is usually associated with various dyslipidemic conditions. Our results show that administration of letrozole induced disordered lipid distribution (significantly lower HDL-C levels and higher TG and TC levels) in the PCOS rats (Figure 6). IR caused an elevated serum level of T and accumulation of visceral fat. This phenomenon is mainly because the body has abnormal glucose consumption, and glucose is converted to lipids in the liver during IR [54]. Excessive androgen can affect metabolic homeostasis in adipose tissue and the liver of PCOS patients [37]. Some research suggests that sensitive characteristics involving elevated TG, TC, TG/HDL, and TC/HDL and decreased TC are valuable markers in reflecting metabolic dysfunction [55, 56]. In this study, we observed that both Rha and Met treatment could reverse these parameters (Figure 6). It indicates that Rha has the capacity to improve metabolism and reproduction.

Oxidative stress has been previously reported as the main characteristic involved in the pathogenesis of PCOS [57]. Antioxidant enzymes act as the first line of defense against oxidative stress by inhibiting the formation of reactive oxygen species (ROS) and preventing damage to lipids and proteins [24]. Enzymatic SOD has an antioxidant effect against superoxide anions that reduces superoxide anion radicals and catalyzes their conversion to H_2_O_2_, which is subsequently converted into H_2_O by glutathione peroxidase (GPx) [58]. There is a high correlation between ROS and reproductive functions [59]. However, overgeneration of ROS may induce many pathological events, including infertility, PCOS, preeclampsia, and endometriosis, in human reproduction [60]. Letrozole-induced PCOS rats have been reported to show serious oxidative damage, indicating an imbalance in the antioxidative/oxidative system, cell membrane destruction, and overgeneration of MDA in the ovaries [41]. Our results strongly suggest that Rha could reduce MDA generation and increase the serum activity of SOD, which protects ovarian tissue against oxidative damage in PCOS rats (Figure 7). Additionally, supplementation with antioxidants is helpful in the management of PCOS, which is associated with improving sex hormones and restoring ovary function [61].

Histomorphological observation revealed that the ovaries displayed cystic expansion and the number of healthy follicles and granular cell layers was reduced in PCOS rats; there was obvious fibrosis in the ovarian tissue (Figures 2(b) and 2(c)). In the current study, granulosa cell degeneration leads to interstitial fibroblast hyperplasia, fibrinogen, and collagen deposition inducing ovarian fibrosis [62]. Studies have shown that TGF-*β* and CTGF were the main factors in promoting the fibrosis of organs [8]. The overexpression of TGF-*β* in various tissues results in substantial fibrosis. In particular, TGF-*β*1, a multifunctional cytokine, is frequently associated with follicular development [63, 64]. The TGF-*β*1/Smad signaling pathway is the critical factor in fibrosis formation and development [65]. In the PCOS group, the protein levels of TGF-*β*1, TGF-*β*R1, p-Smad2, p-Smad3, Smad4, *α*-SMA, collagen I, and CTGF were markedly increased, while Smad7 was notably decreased, indicating the activation of the TGF-*β*1/Smad signaling pathway (Figures 8 and 9). IHC assays presented a significant increase in TGF-*β*1, Smad2, Smad3, and Smad4 expression with a significant reduction in Smad7 expression, confirming this finding (Figure 10). The mature TGF-*β*1 forms a tetramer with TGF-*β*R2, thereby phosphorylating TGF-*β*R1 and then activated TGF-*β*R1 phosphorylated Smad2/Smad3 [10]. Cytoplasmic p-Smad2 and p-Smad3 form a heterotrimer with Smad4, subsequently initiating the transcription of fibrosis-related genes (CTGF, collagen I, and *α*-SMA) in the nucleus [12]. Smad7 can downregulate the TGF-*β*1/Smad signaling pathway by competing with P-Smad2/Smad3 [66]. Kim et al. in their study suggested that the TGF-*β*/Smad signaling pathway was activated in fibrotic kidney disease [67]. CTGF is the downstream response element of TGF-*β*1, and CTGF can, via enhancing the activity of TGF-*β*1, promote fibroblast proliferation [68]. After Rha intervention, the expression of TGF-*β*1/Smad signaling pathway-related proteins was decreased. Besides, Rha treatment significantly increased the expression of PPAR*γ* in the PCOS rats (Figure 9). The transcription factor PPAR*γ* is involved in regulating oxidative stress, glucose regulation, inflammatory response, cell differentiation, and apoptosis [69–71]. In addition, PPAR*γ* has been reported to have strong antifibrotic activity and modulates ECM synthesis and degradation, affects myofibroblasts, inhibits fibrosis factor expression, and blocks the activation of TGF-*β*1/Smads [72]. Taken together, our data proved that Rha treatment could inhibit the activation of the TGF-*β*1/Smad signaling pathway and upregulate PPAR*γ* expression, suggesting Rha can prevent fibrosis.

## 5. Conclusion

In general, the results of this study revealed that Rha modulates sex hormone levels and metabolic disturbances and ameliorates ovarian fibrosis in PCOS rats. Rha is also better than Met at modulating sex hormone levels and inhibiting oxidative stress in PCOS rats. In addition, this is the first study that showed the effect of Rha intervention on the inhibition of the TGF-*β*1/Smad signaling pathway in PCOS rats. Rha could be considered a good choice for patients with PCOS. Future clinical trials need to be conducted to confirm these effects.

## Figures and Tables

**Table 1:** The organ index of the Rha-treated PCOS rats.

Organs	Control	PCOS	PCOS+Rha	PCOS+Met
Ovary	0.043 ± 0.033	0.049 ± 0.002^∗^	0.042 ± 0.002^#^	0.040 ± 0.004^##^
Uterus	0.168 ± 0.011	0.051 ± 0.008^∗∗^	0.072 ± 0.004^##^	0.072 ± 0.001^##^
Heart	0.351 ± 0.019	0.326 ± 0.019	0.338 ± 0.030	0.335 ± 0.020
Liver	3.716 ± 0.213	3.194 ± 0.135^∗∗^	3.176 ± 0.265	3.170 ± 0.239
Spleen	0.250 ± 0.019	0.231 ± 0.021	0.235 ± 0.039	0.232 ± 0.022
Lung	0.470 ± 0.026	0.442 ± 0.022	0.495 ± 0.094	0.472 ± 0.028
Kidney	0.817 ± 0.037	0.772 ± 0.053	0.745 ± 0.0594	0.732 ± 0.059

^∗^ *P* < 0.05 and ^∗∗^*P* < 0.01 vs. the control group; ^#^*P* < 0.05 and ^##^*P* < 0.01 vs. the PCOS group. Values are mean ± SD (*n* = 8).

## Data Availability

All generated and analyzed data used to support the findings of this study are included within the article. The data used to support the findings of this study are available from the corresponding author upon request.
